# Electrophysiological and biochemical evaluations of neuropathy risk in oral levodopa versus levodopa/carbidopa intestinal gel treatment

**DOI:** 10.1055/s-0045-1813243

**Published:** 2025-12-08

**Authors:** Miray Erdem, Bugra Selluncak, Mehmet Balal, Halit Fidanci, Meltem Demirkiran

**Affiliations:** 1 Health Sciences University, Adana City Training and Research Hospital, Department of Neurology, Adana, Türkiye.; 2 Cukurova University, Faculty of Medicine, Department of Neurology, Adana, Türkiye.; 3 Health Sciences University, Adana City Training and Research Hospital, Department of Neurology, Division of Neurophysiology, Adana, Türkiye.

**Keywords:** Levodopa, Carbidopa, Parkinson Disease, Polyneuropathies

## Abstract

**Background:**

The association between levodopa treatment and neuropathy in Parkinson's disease (PD) remains controversial, particularly when comparing the oral and intestinal administration routes.

**Objective:**

To compare the electrophysiological and biochemical changes in patients receiving oral levodopa or levodopa/carbidopa intestinal gel (LCIG) and to determine their association with neuropathy development.

**Methods:**

The current prospective cross-sectional study included 32 PD patients (18 oral and 14 LCIG). Demographic features, disease duration, Hoehn and Yahr (H&Y) stage, Unified Parkinson's Disease Rating Scale (UPDRS) scores, biochemical parameters (vitamin B12, folate, homocysteine), and electrophysiological values were recorded. Nerve conduction studies (NCSs) of the median, ulnar, peroneal, tibial, and sural nerves were performed unilaterally, using reference values from our neurophysiology laboratory.

**Results:**

The LCIG group presented significantly higher doses of levodopa ( *p* < 0.001), levodopa equivalent daily dose (LEDD; *p* < 0.001), and homocysteine levels ( *p* = 0.002) compared with the oral group. Electrophysiological tests revealed significantly reduced motor amplitudes and sensory conduction velocities in the median, ulnar, tibial, and sural nerves in the LCIG group. The median and tibial F-wave latencies were prolonged in the LCIG group. Correlation analyses indicated significant associations involving homocysteine elevation and conduction abnormalities.

**Conclusion:**

Patients receiving LCIG presented higher homocysteine levels and more frequent electrophysiological abnormalities than those on oral treatment, suggesting a higher risk of polyneuropathy. These findings highlight the importance of biochemical monitoring and individualized treatment strategies in advanced PD.

## INTRODUCTION

Parkinson's disease (PD) is a chronic, progressive neurodegenerative disorder characterized by bradykinesia, rigidity, postural instability, and resting tremor. 1 Levodopa remains the most effective pharmacological treatment for PD. 2 3 However, in the advanced stages of the disease, the plasma concentrations of levodopa after oral administration fluctuate considerably, resulting in inconsistent therapeutic effects. 4 Patients with PD also frequently experience gastrointestinal, urinary, cardiovascular, and autonomic disturbances. 5 Sialorrhea, dysphagia, delayed gastric emptying, increased intestinal bacterial load, and defecation dysfunction are some of the most common gastrointestinal manifestations. These symptoms may emerge in the early stages of the disease, and they tend to worsen progressively. Importantly, intestinal dysfunction has been reported 5 to impair the absorption of orally-administered medications, raising concerns about the reliability of the oral treatment in advanced PD.

To address these limitations, levodopa/carbidopa intestinal gel (LCIG) infusion was developed to provide continuous and stable levodopa delivery, thereby reducing motor fluctuations. 6 7 8 Despite these clinical benefits, LCIG therapy requires an invasive procedure, and it has been associated with the occurrence of polyneuropathy, potentially mediated by B-vitamin depletion and elevated homocysteine levels. 9 10 Neuropathy has also been reported in patients receiving long-term oral levodopa, suggesting that both treatment modalities may carry risks. However, the available literature comparing the oral and intestinal administrations remains limited, particularly with respect to biochemical alterations and subclinical electrophysiological changes.

The aim of the present study was to investigate the electrophysiological and biochemical profiles of PD patients receiving oral levodopa or LCIG and to determine their association with neuropathy development.

## METHODS

### Study design and patients

The current prospective, cross-sectional study was approved by the Ethics Committee of the Health Sciences University, Adana City Training and Research Hospital (under protocol no: 3102, dated January 18, 2024). A total of 32 PD patients with a disease duration of at least 5 years were recruited from the Movement Disorders Outpatient Clinic of the Neurology Department between February and May 2024.

The eligible participants were adults (aged ≥ 18 years) who provided written informed consent and had no comorbidities other than PD. The exclusion criteria were refusal to participate, age < 18 years, presence of neuropathic disorders unrelated to PD, comorbidities affecting the neuromuscular system, or the use of medications with potential neuromuscular effects.

Of the 32 patients who met the inclusion criteria, 18 were receiving oral dopaminergic therapy and were classified as the *oral treatment group* . This group included patients treated with levodopa daily (LDD) as well as those on additional dopaminergic agents, for whom the levodopa equivalent daily dose (LEDD) was calculated according to established methods. 11 12 The remaining 14 patients were classified as the *LCIG group* , consisting of individuals treated with LCIG. Demographic characteristics, complete blood counts, and biochemical parameters were collected for all participants. Disease severity was assessed by a movement disorders specialist using the Hoehn and Yahr (H&Y) scale, as well as the motor and non-motor symptom scores of the Unified Parkinson's Disease Rating Scale (UPDRS).

### Neurophysiological tests

Nerve conduction studies (NCSs) were performed using a Sierra Summit electromyography (EMG) unit (Cadwell Laboratories). The NCSs were only performed when the upper extremity temperature exceeded 32 °C and the lower extremity temperature exceeded 31 °C. If these thresholds were not met, the cold extremities were warmed prior to testing. For sensory and motor NCSs, the high-pass and low-pass filter settings were of 20 to 2 kHz and 20 to 10 kHz respectively. Sweep speed and sensitivity were set at 1 ms/division and 10 µV/division for the sensory NCSs, and at 5 ms/division and 2 mV/division for the motor NCSs. Stimulation and recording were performed using surface electrodes, with supramaximal stimulation applied. The NCSs were conducted unilaterally on both the upper and lower extremities. The compound muscle action potentials (CMAPs) of the median, ulnar, posterior tibial, and peroneal nerves were recorded from the abductor pollicis brevis, abductor digiti quinti, abductor hallucis, and extensor digitorum brevis muscles respectively. The distance between the recording electrode and the stimulation point was set at 5 cm for distal median and ulnar motor NCSs, 10 cm for the posterior tibial NCS, and 8 cm for the peroneal NCS. The compound nerve action potentials (CNAPs) of the median and ulnar nerves were recorded antidromically from the second finger–wrist and fifth finger–wrist segments respectively. Sensory nerve conduction velocities were calculated using peak latency. The CMAP and CNAP amplitudes were measured from peak to peak. The minimum latency among ten F-waves was recorded as the F-wave latency. Abnormalities in the NCSs were identified using reference values established in our clinical neurophysiology laboratory.

### Biochemical evaluation

The levels of vitamin B12, folate, and homocysteine were measured in all patients.

### Statistical analysis

The data gathered from the study was uploaded to the IBM SPSS Statistics for Windows (IBM Corp.) software, version 28.0. The categorical variables were expressed as numbers and percentages, while the continuous variables, as mean and standard deviation values. The normality of the continuous variables was assessed using the Shapiro-Wilk test. The Student's *t* -test was used to compare the continuous variables with a normal distribution between the groups, while the Mann-Whitney U test was used for variables that did not follow a normal distribution. The Chi-squared test or the Fisher's exact test was used to analyze the categorical variables. The Monte Carlo exact test was used for small samples. The correlations involving the LEED, the homocysteine, folate, vitamin B12, and levodopa levels, and the electrophysiological parameters of the periferal nerves were assessed using Spearman's correlation test. Values of *p* < 0.05 were considered statistically significant.

## RESULTS

A total of 32 patients were included in the study, with 18 patients in the oral treatment group and 14 in the LCIG group. The mean age of the cohort was of 67.3 ± 6.7 years, and 31.3% (n = 10) of the subjects were female, and 68.8% (n = 22), male.

As shown in Table 1 , there were no significant differences between the two groups with respect to age, disease duration, H&Y stage, UPDRS scores, or serum vitamin B12 and folate levels. In contrast, patients in the LCIG group required significantly higher daily levodopa doses and LEDD compared with those in the oral group ( *p* < 0.001 for both). Moreover, the homocysteine levels were significantly elevated in the LCIG group ( *p* = 0.002).

**Table 1:** Clinical characteristics, total levodopa and homocysteine plasma levels of the study population

Variable		LCIG group (n = 14)	Orally-treated group (n = 18)	*p* -value
Mean age (years)		65.7 ± 6.2	68.6 ± 6.9	0.283
Female sex:* n (%)		4 (28.6)	6 (33.3)	0.773
Mean disease duration (years)		13.0 (5.6)	10.2 (4.4)	0.251
Hoehn and Yahr stage: median (IQR)		4.0 (1.0)	3.0 (1.0)	0.267
UPDRS motor score		34.0 (18.2)	31.5 (10.5)	0.220
Mean levodopa dose (mg)		2,418.7 (671.0)	959.3 (169.8)	< 0.001
Mean LEDD dose (mg)		2,484.7 (668.1)	1334.6 (278.5)	< 0.001
Parkinson's subtype: n (%)	Akinetic-rigid	9 (64.3)	11 (61.1)	0.854
	Tremor dominant	5 (35.7)	7 (38.9)	
Score on the non-motor symptoms scale: median (IQR)		15.0 (4.7)	16.0 (5.0)	0.722
Vitamin B12 level (pg/mL): median (IQR)		170.0 (143.5)	187.0 (141.0)	0.512
Homocysteine level (μmol/L): median (IQR)		29.5 (42.0)	16.0 (15.7)	0.002
Folate level (μg/L): median (IQR)		7.4 (5.1)	6.2 (3.8)	0.639

Abbreviations: IQR, interquartile range; LCIG, levodopa/carbidopa intestinal gel; LEDD, levodopa equivalence daily dose; UPDRS, Unified Parkinson's Disease Rating Scale.

Note: *The Monte Carlo's exact test was used.

Table 2 shows in detail the electrophysiological findings. Compared with the oral group, LCIG patients exhibited significantly lower median motor amplitude, median sensory conduction velocity, and median sensory amplitude ( *p* = 0.041, 0.016, and 0.005 respectively). Ulnar sensory conduction velocity and amplitude were likewise reduced in the LCIG group ( *p* = 0.009 for both). Tibial motor conduction velocity, as well as sural sensory conduction velocity and amplitude, were also significantly lower in the LCIG group ( *p* = 0.011, 0.001, and 0.025 respectively). Furthermore, the median F-wave latency was significantly prolonged in LCIG patients compared with the oral group ( *p* = 0.026). The relationships between biochemical parameters and electrophysiological outcomes are presented in Tables 3 and 4 . In the LCIG group, elevated homocysteine levels showed a moderate negative correlation with sural conduction measures and tibial motor velocity, while vitamin B12 deficiency correlated with reduced tibial conduction. In the oral group, homocysteine levels correlated negatively with peroneal and ulnar latencies, while folate and vitamin B12 levels showed positive associations with conduction values. In addition, levodopa and LEDD levels were negatively correlated with sural sensory amplitude in the LCIG group and positively correlated with median F-wave latency in the oral group.

**Table 2:** Comparison of sensorimotor potentials, amplitudes, and latencies of the median, ulnar, peroneal, tibial, and sural nerves according to treatment groups

Variable	LCIG group (n = 14)	Orally-treated group (n = 18)	*p* -value
Median motor velocity (m/s): median (IQR)	50.1 (6.5)	53.1 (10.5)	0.193
Median motor amplitude (µV): median (IQR)	7.1 (4.3)	13.0 (8.2)	**0.041**
Median motor latency (m/s): median (IQR)	3.4 (0.5)	3.1 (0.9)	0.107
Median sensory velocity (m/s): median (IQR)	34.2 (23.8)	41.0 (9.9)	**0.016**
Median sensory amplitude (µV): median (IQR)	11.3 (18.9)	24.7 (7.5)	**0.005**
Median sensory latency (m/s): median (IQR)	3.4 (2.0)	3.6 (0.3)	0.652
Ulnar motor velocity (m/s): median (IQR)	55.0 (9.6)	55.0 (10.1)	0.653
Mean ulnar motor amplitude (µV)	11.5 ± 4.3	13.5 ± 6.5	0.301
Ulnar motor latency (m/s): median (IQR)	2.5 (0.4)	2.5 (0.3)	0.925
Ulnar sensory velocity (m/s): median (IQR)	30.0 (39.2)	39.0 (8.5)	**0.009**
Ulnar sensory amplitude (µV): median (IQR)	10.2 (23.0)	24.5 (26.5)	**0.009**
Ulnar sensory latency (m/s): median (IQR)	2.9 (3.9)	3.2 (0.4)	0.918
Peroneal motor velocity (m/s): median (IQR)	44.0 (8.4)	49.0 (25.2)	0.281
Peroneal motor amplitude (µV): median (IQR)	1.6 (3.7)	2.3 (2.7)	0.869
Peroneal motor latency (m/s): median (IQR)	4.3 (1.6)	3.7 (3.4)	0.133
Tibial motor velocity (m/s): median (IQR)	40.3 (6.4)	45.1 (15.0)	**0.011**
Tibial motor amplitude (µV): median (IQR)	5.0 (6.1)	9.4 (5.0)	0.168
Tibial motor latency (m/s): median (IQR)	4.9 (1.0)	4.8 (2.2)	0.464
Sural sensory velocity (m/s): median (IQR)	0.0 (35.8)	40.2 (9.2)	**0.001**
Sural sensory amplitude (µV): median (IQR)	0.0 (7.8)	6.6 (2.2)	**0.025**
Sural sensory latency (m/s): median (IQR)	0.0 (3.7)	2.9 (1.0)	0.246
Median F response: median (IQR)	31.3 (11.5)	28.2 (6.2)	**0.026**
Mean tibial F response	47.9 ± 26.7	47.3 ± 22.4	0.376

Abbreviations: IQR, interquartile range; LCIG, levodopa/carbidopa intestinal gel.

Note: Values of *p* in bold indicate statistical significance.

**Table 3:** Correlations between the homocysteine, folate, vitamin B12 and neurographically-impaired nerves

	Homocysteine				Folate				Vitamin B12			
	LCIG group		Orally-treated group		LCIG group		Orally-treated group		LCIG group		Orally-treated group	
	Rho	*p*	Rho	*p*	Rho	*p*	Rho	*p*	Rho	*p*	Rho	*p*
Median motor velocity	−0.475	0.86	−0.283	0.256	0.402	0.154	0.227	0.365	−0.103	0.725	0.296	0.233
Median motor amplitude	−0.135	0.646	0.116	0.647	0.053	0.858	0.050	0.843	0.152	0.604	−0.033	0.896
Median motor latency	−0.115	0.696	−0.103	0.684	−0.090	0.759	0.165	0.514	0.073	0.805	−0.073	0.772
Median sensory velocity	−0.478	0.84	−0.198	0.431	0.309	0.282	−0.155	0.539	−0.200	0.492	0.225	0.369
Median sensory amplitude	−0.458	0.100	0.139	0.582	0.218	0.454	−0.037	0.883	0.278	0.336	0.302	0.224
Median sensory latency	−0.018	0.952	−0.304	0.271	0.274	0.344	0.712	0.003	0.200	0.492	0.027	0.924
Ulnar motor velocity	−0.378	0.183	0.133	0.599	0.535	**0.049**	0.086	0.733	−0.315	0.273	−0.232	0.355
Ulnar motor amplitude	0.221	0.447	0.152	0.547	−0.097	0.741	0.090	0.721	0.176	0.546	0.041	0.871
Ulnar motor latency	−0.088	0.764	−0.481	0.044	0.044	0.881	0.157	0.533	0.456	0.101	0.482	**0.043**
Ulnar sensory velocity	−0.530	0.051	−0.165	0.513	0.236	0.416	0.186	0.460	0.073	0.805	0.470	**0.049**
Ulnar sensory amplitude	−0.424	0.131	0.105	0.677	0.232	0.425	0.106	0.674	0.355	0.212	0.155	0.539
Ulnar sensory latency	0.024	0.934	−0.147	0.587	−0.415	0.140	0.148	0.584	0.685	0.007	−0.232	0.388
Peroneal motor velocity	0.008	0.979	−068	0.794	0.187	0.540	0.137	0.601	−0.537	0.058	0.105	0.689
Peroneal motor amplitude	0.078	0.801	−0.218	0.401	0.328	0.273	0.085	0.746	−0.243	0.424	0.059	0.822
Peroneal motor latency	0.092	0.765	−0.640	0.006	0.053	0.864	0.428	0.086	−0.271	0.370	0.313	0.221
Tibial motor velocity	−0.183	0.531	0.066	0.796	0.464	0.095	−0.095	0.707	−0.640	0.014	0.052	0.839
Tibial motor amplitude	−0.493	0.073	0.079	0.756	0.429	0.126	−0.074	0.770	−0.262	0.366	0.228	0.364
Tibial motor latency	0.351	0.218	0.233	0.353	−0.180	0.537	−0.318	0.198	0.315	0.273	−0.288	0.246
Sural sensory velocity	−0.543	0.045	0.171	0.497	0.136	0.644	−0.089	0.729	0.054	0.855	0.345	0.161
Sural sensory amplitude	−0.507	0.064	0.129	0.609	0.207	0.477	0.004	0.987	0.023	0.938	0.103	0.683
Sural sensory latency	−0.548	0.042	0.461	0.063	0.156	0.594	−0.246	0.341	0.054	0.855	−0.390	0.122
Median F response	−0.273	0.446	0.116	0.680	−0.152	0.676	−0.161	0.566	0.261	0.467	−0.396	0.143
Tibial F response	−0.024	0.947	−0.263	0.386	−0.030	0.934	0.008	0.979	−0.127	0.726	0.132	0.666

Abbreviation: LCIG, levodopa/carbidopa intestinal gel.

Notes: Rho values according to the Spearman's correlation coefficient; *p* -values in bold indicate statistical significance.

**Table 4:** Correlations involving levodopa, LEDD, and neurographically-impaired nerves

	Levodopa				LEDD			
	LCIG group		Orally-treated group		LCIG group		Orally-treated group	
	Rho	*p*	Rho	*p*	Rho	*p*	Rho	*p*
Median motor velocity	−446	0.110	−0.227	0.365	−0.257	0.375	−0.066	0.794
Median motor amplitude	0.031	0.917	0.306	0.218	0.216	0.459	0.353	0.150
Median motor latency	−0.183	0.532	−0.151	0.549	−0.081	0.782	−0.181	0.472
Median sensory velocity	−278	0.336	0.535	**0.022**	−0.376	0.185	0.527	**0.025**
Median sensory amplitude	−431	0.124	0.260	0.298	−0.487	0.078	−0.008	0.974
Median sensory latency	−0.029	0.922	−0.102	0.717	−0.122	0.677	−0.474	0.074
Ulnar motor velocity	−308	0.284	−0.293	0.238	−0.207	0.478	0.150	0.553
Ulnar motor amplitude	0.209	0.472	0.327	0.185	0.370	0.192	−0.139	0.581
Ulnar motor latency	0.057	0.846	0.106	0.675	0.218	0.454	−0.102	0.688
Ulnar sensory velocity	−0.369	0.195	−0.062	0.807	−0.188	0.521	−0.076	0.763
Ulnar sensory amplitude	−0.616	**0.019**	−0.166	0.512	−0.391	0.167	−0.236	0.345
Ulnar sensory latency	0.177	0.545	0.455	0.076	0.206	0.481	0.352	0.181
Peroneal motor velocity	−0.402	0.173	−0.125	0.632	−0.325	0.278	0.160	0.540
Peroneal motor amplitude	−0.425	0.148	−0.238	0.358	−0.284	0.347	0.226	0.383
Peroneal motor latency	0.019	0.950	−0.439	0.078	−0.069	0.822	−0.268	0.299
Tibial motor velocity	−0.402	0.154	−0.111	0.662	−0.297	0.303	0.200	0.425
Tibial motor amplitude	−0.216	0.459	0.223	0.374	−0.128	0.664	0.179	0.478
Tibial motor latency	0.147	0.615	0.074	0.769	0.229	0.431	0.313	0.206
Sural sensory velocity	−0.484	0.079	0.040	0.876	−0.315	0.273	−0.134	0.596
Sural sensory amplitude	−0.576	0.031	−0.031	0.902	−0.443	0.113	−0.145	0.565
Sural sensory latency	−0.505	0.066	0.065	0.805	−0.382	0.178	0.399	0.058
Median F response	−0.321	0.365	0.551	**0.033**	−0.055	0.881	0.346	0.206
Tibial F response	−0.382	0.276	0.071	0.819	−0.442	0.200	0.436	0.137

Abbreviations: LCIG, levodopa/carbidopa intestinal gel; lEDD: levodopa equivalence daily dose.

Notes: Rho values according to the Spearman's correlation coefficient; *p* -values in bold indicate statistical significance.

**Figure 1:**
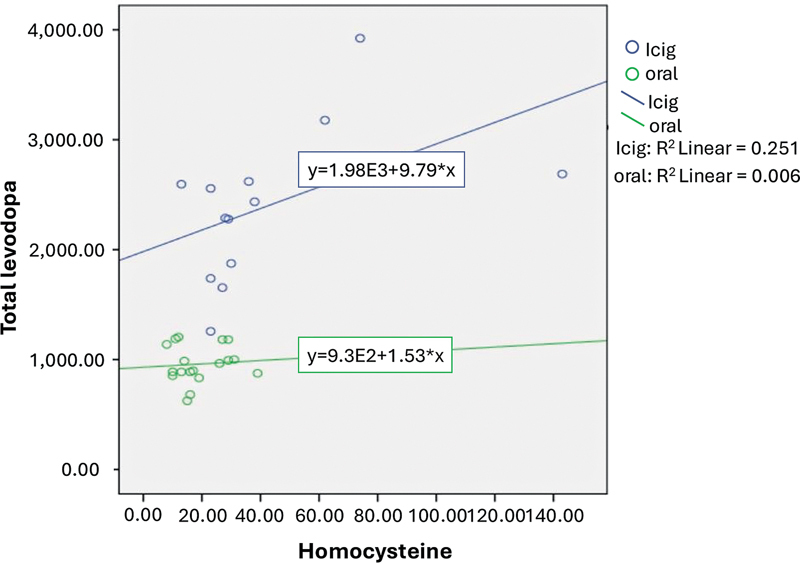
The difference in total levodopa and levodopa equivalence daily dose (LEDD) levels between groups.

Significant differences in daily levodopa dose and LEDD were observed between the two groups ( Figure 1 ).

## DISCUSSION

The most important finding of the current study is that, while a significant increase in levodopa dose, LEDD, and homocysteine level was observed in the LCIG group, patients receiving oral levodopa demonstrated better conduction parameters in electrophysiological tests. Othman et al. 13 showed that LCIG treatment provides a more stable distribution of levodopa and causes fewer fluctuations in its plasma concentrations compared to oral administration, thus ensuring more stable control of motor symptoms through continuous and predictable drug release. The present study highlights that, despite this pharmacokinetic superiority, LCIG use is accompanied by adverse biochemical consequences, including elevated homocysteine levels associated with higher levodopa doses and LEDD requirements. These findings suggest that the clinical benefits of LCIG must be carefully balanced against its biochemical risks.

Loens et al. 14 reported a marked decrease in pyridoxine (vitamin B6) levels and hyperhomocysteinemia in patients receiving LCIG, suggesting that high-dose LCIG negatively affects vitamin B metabolism. Similar biochemical alterations were observed in our LCIG cohort; however, in contrast to the study by Loens et al., 14 our analysis incorporated both biochemical and electrophysiological data. We demonstrated that conduction abnormalities in the LCIG group were consistent with low pyridoxine and elevated homocysteine levels, thereby linking metabolic derangements with neurophysiological impairment. This integrated approach provides stronger evidence for the potential neuropathogenic effects of LCIG therapy.

Jugel et al. 10 demonstrated that axonal neuropathy was more pronounced in LCIG-treated patients, based solely on electrophysiological criteria. While their study emphasized the electrophysiological aspects, the findings of the current expand on this by integrating biochemical parameters such as LEDD and vitamin B6 and homocysteine levels, thus offering a more comprehensive assessment of neuropathy risk. Importantly, our results indicate that conduction abnormalities in the LCIG group are consistent with metabolic derangements, while correlations observed in the orally-treated group suggest that high-dose oral levodopa may also contribute to biochemical instability capable of impairing nerve function.

Taher et al. 15 further demonstrated that pyridoxine deficiency and elevated homocysteine levels in LCIG-treated patients are associated with conduction abnormalities. Our findings are consistent with these results, reinforcing the importance of monitoring both biochemical and neurophysiological parameters in patients undergoing long-term levodopa therapy. Together, these observations highlight the critical role of vitamin B metabolism in neuropathy risk, irrespective of administration route.

Taken together, the results of the current study suggest that, while LCIG provides pharmacokinetic and clinical benefits, its potential for inducing neuropathy via biochemical and electrophysiological pathways must not be overlooked. Importantly, the present study was designed with the consideration that neuropathic findings could arise not only in LCIG-treated patients, but also in those receiving long-term oral levodopa. Although we did not detect significant neurophysiological abnormalities in the oral group, the biochemical correlations observed highlight the need for vigilance in both treatment populations. In general, the main similarity between previous studies and the current is that the high levodopa doses required for LCIG treatment promote elevated homocysteine levels and neuropathy by disrupting vitamin B metabolism. However, the present study differs in its broader scope: rather than focusing solely on pharmacokinetics or biochemical disturbances, we integrated clinical, electrophysiological, and biochemical data to provide a more comprehensive assessment of the effects of LCIG. This multidimensional approach highlights the current study's unique contribution, demonstrating that conduction abnormalities in the LCIG group are closely associated with metabolic derangements, while also showing that correlations in the oral treatment group may indicate a risk of biochemical instability even without overt neurophysiological abnormalities. Importantly, our findings suggest that individualized treatment strategies and close monitoring of vitamin and homocysteine levels, as well as nerve conduction, are essential, particularly for long-term therapy.

### Limitations

The current study has several limitations that should be acknowledged. First, it was conducted at a single center with a relatively small number of participants, which may limit the generalizability of the findings. Second, the NCSs were performed unilaterally; therefore, bilateral variations could not be assessed. Third, small fiber involvement, which is an important contributor to neuropathy, was not evaluated. Finally, patients in the LCIG group received substantially higher LEDD compared with the oral group, which may act as a confounding factor when interpreting the relationship between treatment modality and neuropathy risk.

Despite these limitations, the study provides important comparative data on the biochemical and electrophysiological profiles of PD patients receiving oral versus LCIG therapy, and it highlights the need for larger, multicenter studies including clinical and small fiber assessments.

In conclusion, the present study demonstrated that, while LCIG provides pharmacokinetic advantages and more stable motor symptom control in patients with advanced PD, these benefits are accompanied by significant biochemical and electrophysiological alterations. The elevated homocysteine levels, reduced vitamin B6 metabolism, and nerve conduction abnormalities observed in the LCIG group suggest an increased risk of neuropathy associated with high-dose levodopa exposure. Although no significant electrophysiological abnormalities were detected in the oral levodopa group, biochemical correlations indicate that long-term oral therapy may also contribute to metabolic instability. These findings emphasize the importance of individualized treatment strategies, as well as the need for close biochemical and neurophysiological monitoring in patients receiving chronic levodopa therapy, regardless of the administration route. Further large-scale, longitudinal studies are warranted to better clarify the long-term neuropathic risks and to optimize levodopa treatment regimens in PD.
